# *Eucalyptus torquata* L. flowers: a comprehensive study reporting their metabolites profiling and anti-gouty arthritis potential

**DOI:** 10.1038/s41598-023-45499-0

**Published:** 2023-10-31

**Authors:** Rehab M. S. Ashour, Riham A. El-Shiekh, Mansour Sobeh, Mohamed A. O. Abdelfattah, Marwa M. Abdel-Aziz, Mona M. Okba

**Affiliations:** 1 Department of Pharmacognosy, Faculty of Pharmacy, Cairo University, Cairo, Egypt; 2 Institute of Pharmacy and Molecular Biotechnology, Heidelberg University, Im Neuenheimer Feld 364, 69120 Heidelberg, Germany; 3 College of Engineering and Technology, American University of the Middle East, Egaila, 54200 Kuwait; 4 Regional Center for Mycology and Biotechnology (RCMB), Al-Azhar University, Cairo, 11651 Egypt

**Keywords:** Biochemistry, Drug discovery, Plant sciences

## Abstract

Gouty arthritis is one of the most common metabolic disorders affecting people. Plant based drugs can lower the risk of this health disorder. The anti-gouty potential of *Eucalyptus torquata* flowers methanol extract (ETME) was evaluated in vitro via measuring the inhibitory effects of five pro-inflammatory enzymes; xanthine oxidase (XO), hyaluronidase, lipoxygenase (5-LOX), cyclooxygenases COX-1, and COX-2, in addition to evaluating the inhibition of histamine release, albumin denaturation, membrane stabilization, tyrosinase, and protease inhibitory activities. Also, its antioxidant potential was determined using 2,2-diphenyl-1-picrylhydrazyl (DPPH), 2,2′-Azino-bis(3-ethylbenzothiazoline-6-sulfonic acid (ABTS) radical scavenging assays and ferric reducing power assay (FRAP). HPLC–PDA-MS/MS was used to identify the metabolites in the tested extract. The latter exhibited substantial anti-arthritic properties in all assays with comparable potential to the corresponding reference drugs. HPLC–MS/MS analysis of this bioactive extract tentatively annotated 46 metabolites including phloroglucinols, gallic and ellagic acids derivatives, terpenes, flavonoids, fatty acids, and miscellaneous metabolites. Our study highlights the medicinal importance of *E. torquata* as an anti-gouty candidate and opens new avenues of gouty management.

## Introduction

Gout, also known as gouty arthritis, is a common disease characterized by hyperuricemia, abnormally high level of blood uric acid that leads to the accumulation of monosodium urate crystals in joints causing inflammatory arthritis^1,2^. Gouty arthritis has been associated with the development of other several disorders including cancer, obesity, hyperlipidemia, hypertension, and diabetes^3^. Non-steroidal anti-inflammatory drugs and colchicine are typically utilized in gout treatment, however they come with several adverse effects that impose high demand for safer alternatives^4^.

Several medicinal plants and phytochemicals play a major role in the treatment of a wide array of diseases and pathological conditions, among them oxidative stress related disorders^5–11^. High levels of oxygen reactive species and free radicals end up in a state of cellular oxidative stress, which mediate acute inflammation through cytokines stimulation and pro-inflammatory enzymes activation, such as lipoxygenase, cyclooxygenase, hyaluronidase, tyrosinase and xanthine oxidase^12,13^. Also, denaturation of proteins, membrane stabilization and protease inhibitory activity are well documented causes for inflammation and rheumatoid arthritis. In addition, histamine is well reported as a potent inflammatory mediator^14^. Therefore, the discovery of natural secondary metabolites with pro-inflammatory enzymes inhibiting activity along with potent antioxidant potential would be highly beneficial in controlling and combating gouty arthritis.

*Eucalyptus* (family Myrtaceae) is a well-known genus that is traditionally used to treat a wide array of disorders^15^. *Eucalyptus* plants accumulate various classes of secondary metabolites including phloroglucinols, flavonoids, essential oils, tannins, triterpenes, and oleuropeic acid derivatives^9,16^ with a broad spectrum of pharmacological activities, such as antibacterial, antioxidant, anticancer, antiseptic, and anti-inflammatory potentials^15,17^. *E. torquata* Luehm, known as coral gum, is an endemic tree of Western Australia. Its leaves, stems, and flowers furnished promising antibacterial, antifungal, and anticancer activities^18^.

The aim of this study is to evaluate the potential of *E. torquata* flowers methanol extract in the management of gouty arthritis, by testing its inhibitory activity against several key enzymes related to gout and inflammation. These include xanthine oxidase, hyaluronidase, 5-LOX, COX-1, COX-2, tyrosinase, and protease enzymes. In addition, the potential of *E. torquata* to affect histamine release, albumin denaturation, and membrane stabilization will be explored. Moreover, HPLC–MS/MS will be used to tentatively annotate the secondary metabolites composition of ETME.

## Material and methods

### Plant material and extraction

*Eucalyptus torquata* Luehm. flowers were collected in May 2019 from El-Kobba palace, Cairo, Egypt. Mrs. Therese Labib, Consultant of Plant Taxonomy at the Ministry of Agriculture, Cairo, Egypt authenticated the plant. A voucher specimen (No. 05.06.19.II) was deposited in the Pharmacognosy Department, Faculty of Pharmacy, Cairo University Museum. The air-dried flowers were ground, extracted by maceration in methanol. The combined methanol extract was concentrated under reduced pressure at temperature not exceeding 50 ºC till dryness. Collection of the plant material, complied with the national, institutional, and international legislations and guidelines.

### Chemicals

Methanol, DPPH (2,2-diphenyl-1-picrylhydrazyl), ABTS (2,2′-Azino-bis (3-ethylbenzothiazoline-6-sulfonic acid)), TPTZ (2,4,6-tri(2-pyridyl)-s-triazine), ascorbic acid, hyaluronic acid (human umbilical cord), sodium hydroxide, hyaluronidase (bovine testes), calcium chloride, *p*-dimethylaminobenzaldehyde (PDMAB), and sodium borate were purchased from Sigma-Aldrich (USA). COX (ovine) and LOX inhibitor screening assay kits (Cayman Chemical Company, MI, USA), 96-well cell culture plate (Corning Life Sciences, Lowell, MA, USA), PMA (Sigma-Aldrich, St. Luis, MO, USA), and histamine release assay kit (SPI-Bio, France) were used.

### Biological study

#### In vitro antioxidant activity

DPPH radical scavenging, ABTS radical scavenging and FRAP assays were done according to Dudonne et al.^19^ method minor modifications. The details are mentioned in the supplementary file.

### In vitro anti-inflammatory and anti-arthritic activities

#### COX-1, COX-2, and LOX inhibitory activity

The COX inhibitory activity was assayed using Cayman colorimetric COX (ovine) inhibitor screening assay kit, according to the manufacturer’s instructions. The selectivity index (S.I.) was then calculated as IC_50_ (COX-1)/IC_50_ (COX-2). In vitro 5-lipoxygenase (5-LOX) inhibitory assay was carried out using lipoxygenase inhibitor screening assay kit, according to the manufacturer’s instructions. Diclofenac sodium was used as a reference drug.

#### Albumin denaturation, membrane stabilization and protease inhibitory activities

Albumin denaturation inhibition was evaluated using the previously described method^20^. Membrane stabilization and proteinase inhibitory potentials were evaluated using the previously reported method^21^. Diclofenac was used as the reference drug. All the experiments were performed in triplicate.

#### Xanthine oxidase (XO) inhibitory activity

The XO inhibitory activity was assayed following the protocol reported in^22^. Detailed procedures are described in the supplementary file.

#### Tyrosinase inhibitory activity

Tyrosinase inhibition assay was performed with L-DOPA as substrate adopting the previously described method^23^. Detailed procedures are described in the supplementary file.

#### Hyaluronidase inhibitory activity

Hyaluronidase inhibitory activity of ETME was evaluated spectrophotometrically as reported by Perera et al.^24^ with minor modifications. Detailed procedures are described in the supplementary file.

#### Histamine release inhibitory activity

U937 human monocytes (ATCC, Manassas, VA, USA) were used to study the effect of the extract on histamine release. U937 cells (50,000 cells) were plated in a 96-well plate and treated with various concentration (1000–7.81 µg/mL) of the extract, in presence or absence of 20 nM phorbol myristate acetate (PMA) for 1 h. The cell culture supernatants collected from either untreated control or treated cultures were centrifuged at 10,000 g for 5 min at 4 °C and assessed for the released histamine by the commercially available ELISA kit. Diclofenac was used as the positive control.

### Phytochemical analysis

HPLC–PDA-MS was used to identify the phytoconstituents of ETME using ThermoFinnigan LCQ-Duo ion trap mass spectrometer (ThermoElectron Corporation, Waltham, MA, USA) with an ESI source (ThermoQuest Corporation, Austin, TX, USA)^25,26^. Details of the HPLC system are described in the supplementary file.

#### Virtual screening and drug likeness analysis

Molecular modeling was done via docking the phytoconstituents of ETME to cyclooxygenase 1 (COX-1, PDB code: 5WBE), cyclooxygenase 2 (COX-2, PDB code: 5IKR), 5-lipoxygenase (5-LOX, PDB code: 3V99), and xanthine oxidase (XO, PDB code: 3NVY) using MOE software (MOE2022.v11.18.1) as previously described by Sobeh et al.^27^ to virtually screen the binding mode of the extract’s components in the aforementioned enzymes active site. Moreover, drug likeness analysis was performed by calculating the five molecular descriptors associated with drug likeness and oral bioavailability using the QSAR tool of MOE software.

## Results

**Figure 1:**
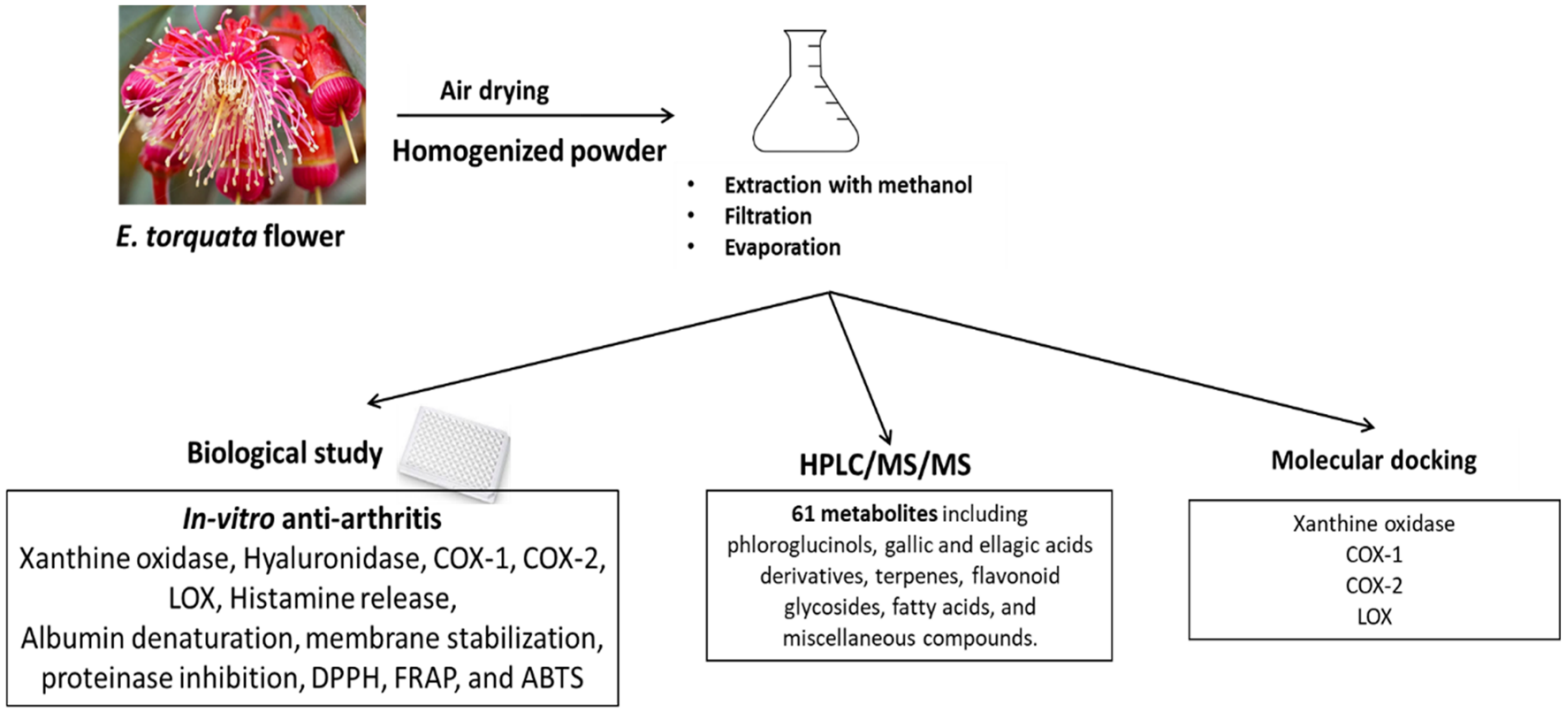
Scheme for extraction, biological evaluation, and chemical analysis of *E. torquata* flowers (ETME)**.**

Gouty arthritis is one of the most common metabolic complaints affecting patients worldwide. Our study traced the potential use of *E. torquata* flowers for the management of hyperuricemia and gout, in correlation with its phytochemical profile. Scheme for extraction, biological evaluation, and chemical analysis of ETME is presented in Fig. 1.

### Biological study

#### In vitro antioxidant activity

ETME exhibited potent antioxidant activity in comparison to ascorbic acid, as revealed by three different assays: DPPH, FRAP, and ABTS, Table 1.

**Table 1:** In vitro antioxidant, anti-inflammatory and antiarthritic potentials of *E. torquata* flower methanol extract (ETME).

Assay	ETME	Reference drugs						
		Ascorbic acid	Celecoxib	Indomethacin	Diclofenac	Sodium aurothiomalate	Kojic acid	Oxypurinol
	IC_50_/EC_50_ (µg/mL)							
DPPH	5.8 ± 0.69	2.2 ± 1.6	–	–	–	–	–	–
FRAP	7.4 ± 0.96	3.9 ± 1.9	-	–	–	–	–	–
ABTS	40.3 ± 1.2	21.4 ± 1.6	-	–	–	–	–	–
COX-1	267.02 ± 1.3	–	248.9 ± 0.78	–	–	–	–	–
COX-2	0.42 ± 0.82	–	0.26 ± 0.99	–	–	–	–	–
SI	635	–	935	–	–	–	–	–
LOX	48.4 ± 1.4	–	–	23.4 ± 0.83	–	–	–	–
Albumin denaturation	26.2 ± 0.76	–	–	–	15.12 ± 1.8	–	–	–
Membrane stabilization	24.4 ± 1.6	–	–	–	17.02 ± 1.3	–	–	–
Protease	31.1 ± 0.86	–	–	–	25.14 ± 1.2	–	–	–
Histamine release	33.2 ± 1.4	–	–	–	17.94 ± 1.4	–	–	–
Hyaluronidase	31.06 ± 0.58	–	–	–	–	20.03 ± 0.96	–	–
Tyrosinase	60.8 ± 1.7	–	–	–	–	–	52.0 ± 0.98	–
Xanthine oxidase	22.2 ± 1.3	–	–	–	–	–	–	14.7 ± 0.93

All experiments were carried out in a triplicate manner and values are expressed as mean ± SD. SI is COX selectivity index calculated as IC_50_ (COX-1)/IC_50_ (COX-2).

#### In vitro anti-inflammatory and anti-arthritic activities of *E. torquata* flower methanol extract (ETME)

ETME inhibited COX-1, COX-2 and 5-LOX with comparable potential to the reference drugs. It also showed excellent selectivity towards COX-2 enzyme, Table 1. Anti-gouty arthritic activity of ETME was tested in vitro via evaluating its potential towards albumin denaturation, membrane stabilization, histamine release, and its inhibitory potential against protease, xanthine oxidase, tyrosinase, and hyaluronidase enzymes. ETME showed very promising activity in all tested assays, Table 1**.**

### Phytochemical analysis

The HPLC–MS analysis of ETME allowed the tentative identification of 46 plant metabolites belonging to several phytochemical classes. These include phloroglucinols, gallic and ellagic acid derivatives, terpenes, flavonoid glycoside, fatty acids, and miscellaneous metabolites. Each metabolite observed molecular weight, fragment ions, and its identity is recorded in Table 2**.** Identification of the peaks were based on comparing their spectral masses with the data reported in the previous literature on Genus *Eucalyptus*^6,9,16^.

**Table 2:** Secondary metabolites identified in *E. torquata* flower using HPLC–PDA-MS.

Identification	[M-H]^-^	Main fragments	References
**Phloroglucinols**			
Grandinol	251	249, 207	^28^
Jensenone	265	250, 193, 165	^6^
Euglobal T1, IIb, IIc, or la1	385	249, 207	^29^
Euglobal G1-10 isomers	385	249, 207	^29^
Euglobal In3, Iva, III, V, or VII	453	385, 249, 207	^29^
Macrocarpal A, B, H, K, E or D	471	249, 207	^16^
Macrocarpal AM1/N	485	207	^16^
Macrocarpal I/J	489	471, 265, 249	^16^
Sideroxylonal A, B, or C	499	471, 249, 207	^9^
Grandinal	499	249, 207	^9^
FPC (1)	925	893, 497, 249	^30^
FPC (2)	653	471, 249, 207	
FPC (3)	635	489, 249, 207	
FPC (4)	403	375, 249, 207	
FPC (5)	907	471, 249, 207	
FPC (6)	721	471, 385, 249	
FPC (7)	635	453, 249, 207	
FPC (8)	703	453, 249, 207	
FPC (9)	857	453, 249, 207	
FPC (10)	607	453, 250, 207	
**Gallic and Ellagic acids derivatives**			
Methyl gallate	183	183, 169, 125	^31^
Ellagic acid	301	301, 257, 229	^31^
Methylellagic acid	315	315, 301, 169	^31^
Methyldigallic acid	335	183, 169, 124	^31^
Methyldigallic acid gallate	487	335, 183, 169	^31^
Tetra-*O*-galloyl-glucose	787	635, 465, 169	^15^
Digalloyl-*O*-methyl-glucose	497	313, 271, 169	^15^
Trigalloylglucose	635	477, 301, 169	^15^
Galloyl glucose	331	316, 169	^15^
**Terpenes**			
Nor triterpene	453	385, 249	^16^
Eucalyptanoic acid	453	385, 207	^16^
Ursolic/betulinic acid	455	369, 249, 207	^16^
Hydroxy ursolic acid/Hederagenin	471	385, 249	^16^
Eucalyptic/eucalyptolic acid	647	471, 249, 207	^16^
Lactone of asiatic acid	485	457, 439, 425	^16^
Arjunolic acid	487	458, 249, 207	^16^
Globulusin A	483	425, 249, 165	^32^
**Flavonoid glycosides**			
Kaempferol-rhamnosylrutinoside	739	595, 285, 237	^33^
Quercetin *O*-rhamnosylrutinoside	755	593, 301, 279	^34^
Quercetin-*O*-glucoside-gallate	615	463, 301	^31^
Isorhamnetin *O*-rhamnosylrutinoside	769	623, 315, 271	^33^
Isorhamnetin *O*-diglucoside	639	315, 301	^31^
**Fatty acids**			
Hydroxy tetracosanoic acid	383	365, 325, 299	^16^
Hydroxy octadecadienoic acid C18:2	295	277, 171, 113	^16^
**Miscellaneous**			
Eucalmaidin C	525	463, 239, 197	^35^
Glucosyl-dihydroxy-isobutylchromone	395	233, 275, 189	^35^

Results revealed the high abundance of ***phloroglucinols*** in ETME; 34 phloroglucinols. Eighteen phloroglucinol-sesquiterpene adducts were tentatively identified. Out of which, five signals with fragmentation pattern; *m/z* 471, *m/z* 249 and *m/z* 207 typical to that reported for macrocarpals^16^; they were annotated as macrocarpals isomers. In addition, ten signals were also detected and identified as euglobals through their intense peak at *m/z* 249 and less intense peak at *m/z* 207 as previously reported^16,30^. Moreover, four dimeric phloroglucinols were identified as sideroxylonal A/B/C and grandinal. They were characterized by the presence of molecular ion peak at *m/z* 499 and daughter ions at *m/z* 471, *m/z* 249 and *m/z* 207. All these metabolites were previously reported in *E. sideroxylon* leaves and flowers^9,16^. Other phloroglucinols, formylated phloroglucinol compounds (FPC 1–10), were tentatively characterized by comparing their masses with the previously reported data^30,32^.

Nine gallic and ellagic acid derivatives were detected. Fragments of galloyl moiety (*m/z* 169) and product ions due to its loss [M–H - 169]^−^ were detected in the mass chromatogram of the corresponding peaks.

Regarding terpenes, the presence of molecular ion peak at m/z 455 and daughter ions at *m/z* 249 and *m/z* 207 allowed the tentative identification of ursolic/betulinic acid. The [M–H]^−^ *m/z* 471 exceeds that of ursolic/betulinic acid by 16 Da, which means extra hydroxyl group, this leads to its identification as hydroxy ursolic acid/ hederagenin.

Five flavonoid glycosides; kaempferol, quercetin, and isorhamnetin derivatives have been tentatively identified through their fragmentation pattern that showed the presence of *m/z* 285, 301, and 315, respectively. This fragmentation pattern showed the loss of rhamnose (*m/z* 146), glucose (*m/z* 162), rutinoside (*m/z* 308), and gallate (*m/z* 152) fragments that appeared independently to be cleaved from the main structure.

Two fatty acids were tentatively identified. Their fragmentation is consistent with the previously reported data^16^. The two fatty acids are hydroxy tetracosanoic acid and hydroxy octadecadienoic acid that showed molecular ion peaks at *m/z* 383 and *m/z* 295, respectively.

Two miscellaneous compounds; one oleuropic acid derivative, eucalmaidin C, and one phenolic compound, glucosyl-dihydroxy-isobutylchromone, were also identified in ETME.

### Virtual screening and drug likeness analysis

We have previously studied the binding affinity of several organic acids, phenolics and flavonoids towards some of the prominent pro-inflammatory enzyme targets by the aid of the molecular docking computational tool^25,27^. Herein, we docked 35 phloroglucinols, the most abundant identified class of compounds, to COX-1, COX-2, 5-LOX, and XO Table 3, so that we could virtually investigate their binding affinity and blocking potential towards these key enzymes mediating the inflammation process. This would help identify the key compounds, which could contribute to the extracts’ inhibitory potential against these target proteins and present them as leads for developing novel drug candidates of natural origin. Regarding COX-1 and COX-2 enzymes, it was observed that the docked phloroglucinols showed generally a very good binding affinity to both target enzymes, however they showed much better affinity towards COX-2, which confirms their COX-2/COX-1 selectivity as revealed in the in vitro assays. Sideroxylonal C showed the best affinity (minimum docking score) to COX-1 with a docking score of − 14.23 kcal/mol. However, macrocarpal I showed the highest affinity towards COX-2 with a docking score of − 7.4 kcal/mol and retained a score of − 10.99 kcal/mole towards COX-1, which indicates high selectivity towards COX-2. Docking to 5-LOX revealed appreciable binding affinity of the docked phloroglucinols at the binding site of the target enzyme reflected by the docking score values that ranged from − 18.00 to − 10.67 kcal/mol. The best binding affinity was shown by sideroxylonal B. As for XO, the binding affinity showed by the phloroglucinol derivatives docked into this target enzyme was not as good as it was in the other three targets, as the docking score ranged only from − 7.87 to − 6.03 kcal/mol. Sideroxylonal B was the best docked compound with the least minimum docking score of − 7.87 kcal/mol. It afforded two hydrogen bonding interactions with Ser876 and Val1011, neither of which was reported by the co-crystallized inhibitor quercetin. In view of these results, we could conclude that the extracts’ compounds are most likely targeting COX/LOX enzymatic pathway.

**Table 3:** Docking scores of the identified phloroglucinols from *E. torquata* flowers.

Compound name	Function score (Kcal/mole)			
	5-LOX	COX-1	COX-2	XO
Macrocarpal I	− 14.55	− 10.99	− 17.40	− 7.33
Sideroxylonal B	− 18.00	− 11.09	− 17.32	− 7.87
Sideroxylonal C	− 15.33	− 14.23	− 16.62	− 7.02
Macrocarpal J	− 13.31	− 12.46	− 16.28	− 6.94
Euglobal G7	− 10.81	− 9.70	− 15.37	− 6.49
Macrocarpal K	− 13.71	− 12.31	− 15.24	− 7.55
Macrocarpal H	− 14.43	− 10.43	− 15.13	− 6.08
Macrocarpal N	− 13.32	− 12.34	− 15.04	− 6.66
Euglobal Ia1	− 12.42	− 9.24	− 14.91	− 6.89
Macrocarpal L	− 13.19	− 10.67	− 14.74	− 7.00
Grandinal	− 15.16	− 13.92	− 14.59	− 7.40
Macrocarpal D	− 12.80	− 7.43	− 14.29	− 6.43
Euglobal III	− 12.70	− 11.22	− 13.92	− 6.03
Euglobal IIC	− 10.08	− 9.96	− 13.86	− 6.79
Euglobal V	− 10.93	− 12.44	− 13.80	− 6.49
Macrocarpal A	− 16.14	− 10.45	− 13.29	− 6.76
Eucalyptal E	− 15.50	− 10.16	− 13.27	− 7.02
Euglobal G3	− 12.05	− 9.30	− 13.19	− 7.30
Euglobal G6	− 12.00	− 10.68	− 13.10	− 7.41
Macrocarpal K	− 14.38	− 11.14	− 13.01	− 7.14
Euglobal IIb	− 12.97	− 10.79	− 12.80	− 7.59
Jensenone	− 11.42	− 8.85	− 12.75	− 7.03
Sideroxylonal A	− 17.42	− 12.29	− 12.55	− 7.06
Euglobal Iva	− 13.02	− 9.16	− 12.51	− 7.18
Euglobal In-3	− 12.01	− 11.60	− 12.49	− 6.57
Euglobal G2	− 11.91	− 11.58	− 12.31	− 6.88
Euglobal G9	− 11.23	− 9.04	− 12.29	− 7.47
Macrocarpal B	− 14.25	− 10.39	− 11.88	− 6.59
Euglobal G8	− 11.96	− 10.66	− 11.63	− 6.94
Euglobal G4	− 11.37	− 8.94	− 10.85	− 7.11
Euglobal T1	− 11.53	Failed	− 10.80	− 7.18
Grandinol	− 10.67	− 9.69	− 10.38	− 6.33
Euglobal VII	− 10.97	− 9.39	− 10.36	− 7.70
Euglobal G1	− 11.73	Failed	− 10.04	− 7.12
Euglobal G10	− 11.58	− 7.46	− 9.42	− 7.33

We also investigated the drug likeness potential of the phloroglucinol derivatives identified in the extract by measuring the descriptors associated with drug likeness and oral bioavailability, namely the molecular weight, number of H-bond acceptors (lip_acc), number of H-bond donors (lip_don), and the partition coefficient (logP(o/w)). Among the investigated compounds, only 8 showed to have drug likeness potential as they obeyed Lipiniski’s rule of five, the most common rule used by medicinal chemists to check the oral bioavailability of newly developed drug candidates, Table 4. Interestingly, these compounds included sideroxylonal C and macrocarpal I, which showed the best binding affinity towards COX-1 and COX-2 enzymes, respectively and sideroxylonal B as well that was the best docked compound into both 5-LOX and XO enzymes. These three compounds, thus, can be very promising lead hits for developing novel anti-inflammatory agents of natural origin.

**Table 4:** Drug likeness analysis of phloroglucinol derivatives identified in *E. torquata.*

Compound name	Weight	lip_acc	lip_don	logP(o/w)	lip_druglike	lip_violation
Macrocarpal I	490.64	7	5	5.76	1	0
Sideroxylonal B/C	500.50	10	5	5.03	1	1
Euglobal G7	386.49	5	2	4.37	1	0
Macrocarpal K/H	472.62	6	4	5.81	1	1
Macrocarpal N	486.60	7	3	4.69	1	1

## Discussion

Several studies have been conducted to discover plant-derived natural drugs. We herein report the effective role of *E. torquata* flowers in the treatment of gouty arthritis. A close relationship has been highlighted between uric acid and oxidative stress, where a large amount of reactive oxygen species is produced along with uric acid production. Antioxidants are considered of great importance in the management of hyperuricemia^36^. Also, oxidative stress plays a key role in the pathogenesis of gout and is responsible for a series of inflammatory pathways^37,38^.

Comparable antioxidant properties were reported for several *Eucalyptus* species; *E. camaldulensis*^39,40^, *E. globulus* leaves and bark^41,42^ and *E. sideroxylon* bark^43^. In addition, several Myrtaceae plants are rich in polyphenolics such as *Eugenia uniflora*, *Syzygium samarangense, Syzyium jambos*, *Syzygium aqueum*^44–47^, and *Callistemon citrinus*^48^.

It is well documented that pro-inflammatory enzymes play an essential role in inflammation pathogenesis through different pathways. So, cyclooxygenases and lipoxygenases inhibition are considered as targets for the management of oxidative stress associated diseases and inflammation that contribute in the amelioration of gouty inflammation^24^. It was reported that the selective COX-2 inhibitors that showed 2–100 fold difference in the concentrations needed to inhibit COX-2 versus COX-1, decreases prostaglandins levels at the inflammatory sites only and has no effect on gastric mucosal prostaglandin levels^49^. Our results are in accordance with that reported about the inhibition potential of several *Eucalyptus* species oils and extracts against cyclooxygenases and lipoxygenases enzymes^50–52^.

Denaturation of tissue proteins is well documented in the pathogenesis of inflammatory events like arthritis. The protection against protein denaturation is one of NSAIDs mechanisms of action. In addition, red blood cell hemolysis inhibition may provide insights into the process of inflammation. Stabilization of the red blood cell membranes may delay or prevent the lysis and the consequent cytoplasmic contents release and the inflammatory response. So, plants that can interfere with these responses could be very useful for developing novel anti-arthritic drugs^53^. Natural extracts from other *Eucalyptus* species produced similar albumin denaturation and cell membrane stabilization activities including *E. obliqua* leaves^54^, *E. sideroxylon* bark^9^ and *E. globulus* leaves^55^.

Moreover, during inflammation, leukocytes release lysosomal enzymes, including proteases, triggering further tissue damage and consequent inflammation. Proteinases have been related to arthritic reactions, where a significant level of protection was provided by inhibitors of proteinase enzyme. The observed protease inhibitory potential of ETME is in accordance with the reported data about the proteinase inhibitory activity of other *Eucalyptus* species^9,56^.

Regarding histamine, it is a very important mediator in inflammatory conditions. The synovial fluids from patients with acute gout have significantly high levels of histamine^57^. In addition, hyaluronidase enhances mast cells degranulation and releases inflammatory mediators leading to several pathological conditions including arthritis^24^. Tyrosinase enzyme, on the other hand, is a multifunctional, widely distributed enzyme in nature, which when accrued in excess, melanin results in hyperpigmentation disorders, Parkinson’s disease, and others oxidative stress disorders like arthritis^58^. Interestingly, *E. globulus* extract was previously reported to inhibit histamine release, hyaluronidase and tyrosinase enzymes, which comes in agreement of our data in this study^59–61^.

The oxidation xanthine and hypoxanthine to uric acid is catalyzed by xanthine oxidase^62^. Hyperuricemia is caused by the over expression of xanthine oxidase resulting in high levels of uric acid that contribute to the deposition of monosodium urate in the joint cavity, which in turn activates inflammatory cytokines, leading to gouty arthritis. Therefore, keeping xanthine oxidase and serum uric acid levels within normal range is very important in treating or ameliorating this distressfully agonizing condition^63^. Noteworthy, *Eucalyptus deglupta* was reported to inhibit xanthine oxidase^64^, which, along with our results, confirm the value of this genus in managing inflammation and gouty arthritis.

The secondary metabolites identified in ETME are responsible for its newly explored anti-hyperuricemic and anti-inflammatory potentials. Ursolic acid, for instance, has reported potent hypouricemic activity and XO inhibitory activities^65^. In addition, several studies have reported flavonoids as potent inhibitors of COX, 5-LOX and XO enzymes^66–68^. Furthermore, methyl gallate was reported to possess not only xanthine oxidase inhibitory activity but it also suppressed superoxide anion generated by XO^69^ in addition to its potent COX-I, COX-II and 5-LOX inhibitory activities^70,71^. Ellagic acid was also reported to strongly inhibit XO^72^ and scavenge DPPH stable radical^73^. Phloroglucinols and their derivatives have been reported for their anti-inflammatory and antioxidant activities^74–76^.

The extensively explored *E. torquata* flowers potential, herein, for the management of gouty arthritis suggests its future deep investigation at in vivo and clinical levels for the discovery of novel gouty arthritis drugs. Further chemical investigation on the plant to isolate its bioactive constituents and drug development is in demand as well.

## Conclusion

*Eucalyptus torquata* flowers extract showed potent in vitro anti-gouty activity with remarkable selective COX-2 inhibitory potential. Several secondary metabolites belonging to different phytochemical classes were identified in the extract. The most abundant was the phloroglucinols. In view of our in-silico results, some of the identified metabolites from *E. torquata* flowers can be considered as novel leads for the discovery and development of drug candidates of natural origin that could be used to manage and combat gouty arthritis.

### Supplementary Information

Supplementary Information.

## Data Availability

The datasets used and/or analyzed during the current study are available from the corresponding author on reasonable request.
