# Supplementary material for: Eucalyptus torquata L. flowers: a comprehensive study reporting their metabolites profiling and anti-gouty arthritis potential
Source: Sci Rep. 2023 Oct 31;13:18682. doi: 10.1038/s41598-023-45499-0 (PMC10618445; doi:10.1038/s41598-023-45499-0)
Supplement: Supplementary file 1 — Supplementary Information. [file 41598_2023_45499_MOESM1_ESM.docx]

***Eucalyptus torquata* L. Flowers: A Comprehensive Study Reporting Its Metabolites Profiling and Anti-****Gouty Arthritis Potential**

Rehab M.S. Ashour ^1,#^, Riham A. El-Shiekh ^1,#^, Mansour Sobeh ^2^, Mohamed AO Abdelfattah ^3^, Marwa M. Abdel-Aziz ^4^, Mona M. Okba ^1*^

^1^ Department of Pharmacognosy, Faculty of Pharmacy, Cairo University, Cairo, Egypt.

^2^ Institute of Pharmacy and Molecular Biotechnology, Heidelberg University, Im Neuenheimer Feld 364, 69120 Heidelberg, Germany

^3^ College of Engineering and Technology, American University of the Middle East, Kuwait

^4^ Regional Center for Mycology and Biotechnology (RCMB), Al-Azhar University, Cairo 11651, Egypt

***Corresponding author**

Email: mona.morad@pharma.cu.edu.eg

**Material and Methods**

***Biological study***

***In vitro antioxidant activity***

*DPPH radical scavenging assay*

Briefly, 20 μL of the diluted concentrations of ETME/ standard (0.1575 - 1 mg/mL) were added to 200 μL of 2,2-diphenyl-1-picrylhydrazyl (DPPH) in a 96 well-plate and the absorbance was measured at 517 nm after incubation for 30 min [1] using ascorbic acid as standard. EC_50_ was determined using the equation; [(Ac – As) / Ac] × 100. In which, Ac was the control absorbance (DPPH solution without the tested sample) while As was the sample absorbance (DPPH solution in addition to the extract or standard). The EC_50_ is the sample concentration that produces 50% inhibition of absorbance at the above-mentioned wavelength.

*ABTS radical scavenging assay*

Briefly, 200 µL of the reaction mixture containing 10 µL extract/ standard at different concentrations (0.1575 - 1 mg/mL) was added to 2,2′-Azino-bis(3-ethylbenzothiazoline-6-sulfonic acid (ABTS) solution (190 µL) then measured at 734 nm in 96 well-plates. The ABTS solution was composed as follows; 5 mL of 14 mM ABTS dissolved in methanol added to 5 mL solution of 4.9 mM potassium persulfate, and subsequently stored for 16 h at room temperature in dark. This solution was diluted with methanol till the absorbance was 0.700. The EC_50_ was calculated and the standard used was ascorbic acid [1].

*FRAP assay.*

The ferric reducing power of the extract was determined using the method reported previously by Dudonne, *et al*., 2009 [1]. The reduction is monitored by measuring the change of absorbance at 593 nm. The working FRAP reagent was freshly prepared by mixing 10 volumes of 300 mM acetate buffer, pH 3.6, with 1 volume of 10 mM TPTZ (2,4,6-tri(2-pyridyl)-s-triazine) in 40 mM hydrochloric acid and with 1 volume of 20 mM ferric chloride. A standard curve was prepared using various concentrations of FeSO_4_ .7H_2_O. All solutions were used on the day of preparation. A 100 µL of the sample solution and 300 μL of deionized water were added to 3 mL of freshly prepared FRAP reagent. The reaction mixture was incubated in a water bath for 30 min at 37 °C. The difference between sample absorbance and blank absorbance was calculated and used to calculate the FRAP value. All measurements were done in triplicate and the EC_50_ was calculated. Ascorbic acid is used as standard.

***In vitro anti-inflammatory and anti-arthritic activities***

*Xanthine oxidase (XO) inhibitory activity*

The XO inhibitory activity was assayed following the protocol reported in [2]. Five hundred µL of different concentrations of ETME/ standard (10- 100 µg/mL) were added to the enzyme solution (1.3 mL of phosphate buffer at concentration of 65 mM and pH 7.4 containing 0.28 U/mL of XO). Then the mixture was incubated for 10 min at 30 ℃. Next, 1.4 mL of the substrate xanthine (0.6 mM) was added to the mixture. The absorbance was measured at every minute for 10 min at 295 nm. Allopurinol was used as the standard drug. The percentage inhibition of XO was calculated as: % inhibition = (1 – Absorbance of sample/ Absorbance of blank) x 100. The IC_50_ value was calculated from the calibration curve.

*Tyrosinase inhibitory activity*

Tyrosinase inhibition assay was performed with L-DOPA as substrate adopting the method reported in [3]. In a 96-plate, 685 μL of phosphate buffer (0.05 M, pH 6.5), 15 μL of tyrosinase enzyme (2500 U /mL), 200 μL of ETME (7.81–1000 µg/mL) and 100 μL of 5 mM/L L-DOPA were added. The absorbance was immediately monitored at 492 nm. Kojic acid was used as the positive control. The assay was made in triplicate. The percentage inhibition was calculated as follows: % of inhibition = (A_control_/A_tested sample_)/ A_control_) x 100. Where, A_control_ is the absorbance without the addition of the inhibitor and A_sample_ is the absorbance after adding the standard.

*Hyaluronidase inhibitory activity*

Hyaluronidase inhibitory activity of ETME was evaluated spectrophotometrically as reported by Perera *et al*., 2018 with minor modifications [4]. The tested sample was assayed at the concentration range of 7.81–1000 µg/mL. ETME/ standard (50 μL) was incubated with hyaluronidase enzyme solution (10 μL) at 37 °C for 10 min followed by the addition of calcium chloride (12.5 mM, 20 μL) and re-incubation at 37 °C for 10 min. Sodium hyaluronate (50 μL) was added to the reaction mixture and incubated at 37 °C for 40 min followed by the addition of sodium hydroxide (0.9 M, 10 μL) and sodium borate (0.2 M, 20 μL) before incubation at 100 °C for 3 min. PDMAB (50 μL, 67 mM) was added to the reaction mixture and finally incubated at 37 °C for 10 min. Absorbance was measured at 585 nm. The percentage of enzyme inhibition was calculated as follows:

% of inhibition = (A_control_/A_tested sample_)/ A_control_) x 100.

Where A_control_ is the absorbance without addition of the inhibitor and A_sample_ is the absorbance after adding the extract/standard. The IC_50_ value, the concentration giving 50% inhibition of hyaluronidase activity, was determined by interpolation using the concentration-response curves. Sodium aurothiomalate was used as the reference standard.

***Phytochemical analysis***

HPLC-PDA-MS was used to identify the secondary metabolites of ETME using ThermoFinnigan LCQ-Duo ion trap mass spectrometer (ThermoElectron Corporation, Waltham, Ma, USA) with an ESI source (ThermoQuest Corporation, Austin, Tx, USA) [5]. ThermoFinnigan HPLC system using a Discovery HS F5 column (15 cm × 4.6 mm ID, 5 µm particles, Sigma-Aldrich Co Steinheim, Germany) was used in the study. Water (HPLC grade) and acetonitrile (ACN) (Sigma-Aldrich GmbH, Germany) (0.1 % formic acid each) were used as a mobile phase. At 0 min, ACN was 5% and increased to 30% in 60 min and then increased again to 90% in the net 30 min at 1 mL/min with a 1:1 split before the ESI source. Autosampler surveyor ThermoQuest was utilized to inject the sample and the system was controlled by Xcalibur software (Xcalibur^TM^ 2.0.7, Thermo Fischer Scientific, Waltham, Ma, USA). The ions were detected in a full scan mode and mass range of 50–2000 *m/z* in the negative ionization mode [6].

**References:**

1. Dudonne, S., et al., *Comparative study of antioxidant properties and total phenolic content of 30 plant extracts of industrial interest using DPPH, ABTS, FRAP, SOD, and ORAC assays.* Journal of agricultural and food chemistry, 2009. **57**(5): p. 1768-1774.

2. Lima, R.d.C.L., et al., *Effects of extracts of leaves from Sparattosperma leucanthum on hyperuricemia and gouty arthritis.* Journal of Ethnopharmacology, 2015. **161**: p. 194-199.

3. Di Petrillo, A., et al., *Tyrosinase inhibition and antioxidant properties of Asphodelus microcarpus extracts.* BMC complementary and alternative medicine, 2016. **16**(1): p. 1-9.

4. Perera, H.D.S.M., et al., *In vitro pro-inflammatory enzyme inhibition and anti-oxidant potential of selected Sri Lankan medicinal plants.* BMC complementary and alternative medicine, 2018. **18**(1): p. 271.

5. Sobeh, M., et al., *Albizia anthelmintica: HPLC-MS/MS profiling and in vivo anti-inflammatory, pain killing and antipyretic activities of its leaf extract.* Biomedicine & Pharmacotherapy, 2019. **115**: p. 108882.

6. El-Hawary, S.S., et al., *HPLC-PDA-MS/MS profiling of secondary metabolites from Opuntia ficus-indica cladode, peel and fruit pulp extracts and their antioxidant, neuroprotective effect in rats with aluminum chloride induced neurotoxicity.* Saudi Journal of Biological Sciences, 2020. **27**(10): p. 2829-2838.
